# Unilateral Tensor Fascia Suralis: A Rare Type Ia Variant and Its Clinical Implications

**DOI:** 10.7759/cureus.83277

**Published:** 2025-04-30

**Authors:** Sayan Biswas, Karan Kumar, Praisy Joy, Manisha R Gaikwad

**Affiliations:** 1 Department of Anatomy, All India Institute of Medical Sciences, Bhubaneswar, IND

**Keywords:** accessory muscle, leg, popliteal fossa, tensor fascia suralis, thigh

## Abstract

Tensor fascia suralis is a rare supernumerary muscle in the posterior thigh and leg, with a male preponderance as reported in the literature. We present a unique Type Ia variant discovered during cadaver dissection of the left lower limb, originating from the long head of the biceps femoris and terminating at the medial head of the gastrocnemius. Unlike previously reported cases, this variant exhibited two distinct fleshy muscle bellies at origin and insertion, separated by an intermediate tendon. Both bellies received innervation from the tibial nerve, with the upper belly supplied directly via the sciatic nerve and the lower belly via a branch penetrating the gastrocnemius. This anomaly has significant clinical implications, potentially mimicking soft tissue tumors on imaging or contributing to nerve compression syndromes. Awareness of such variants is crucial for accurate diagnosis and optimizing surgical outcomes in posterior thigh and leg procedures.

## Introduction

The tensor fascia suralis (TFS) is a rare supernumerary muscle located in the lower posterior thigh and leg. It usually originates from one of the hamstrings and inserts into the gastrocnemius, calcaneal tendon, or the crural fascia, and hence is also called ischioaponeuroticus [1]. With a prevalence rate of 1.27%, the presence of this muscle is often incidental, discovered during dissections or imaging studies, more commonly in men. It is mostly supplied by the tibial nerve, which may be compressed. TFS acts as a weak flexor of the knee joint when present. Anatomical variations of this kind may have implications in surgical procedures and clinical imaging [2].

## Case presentation

**Figure 1:**
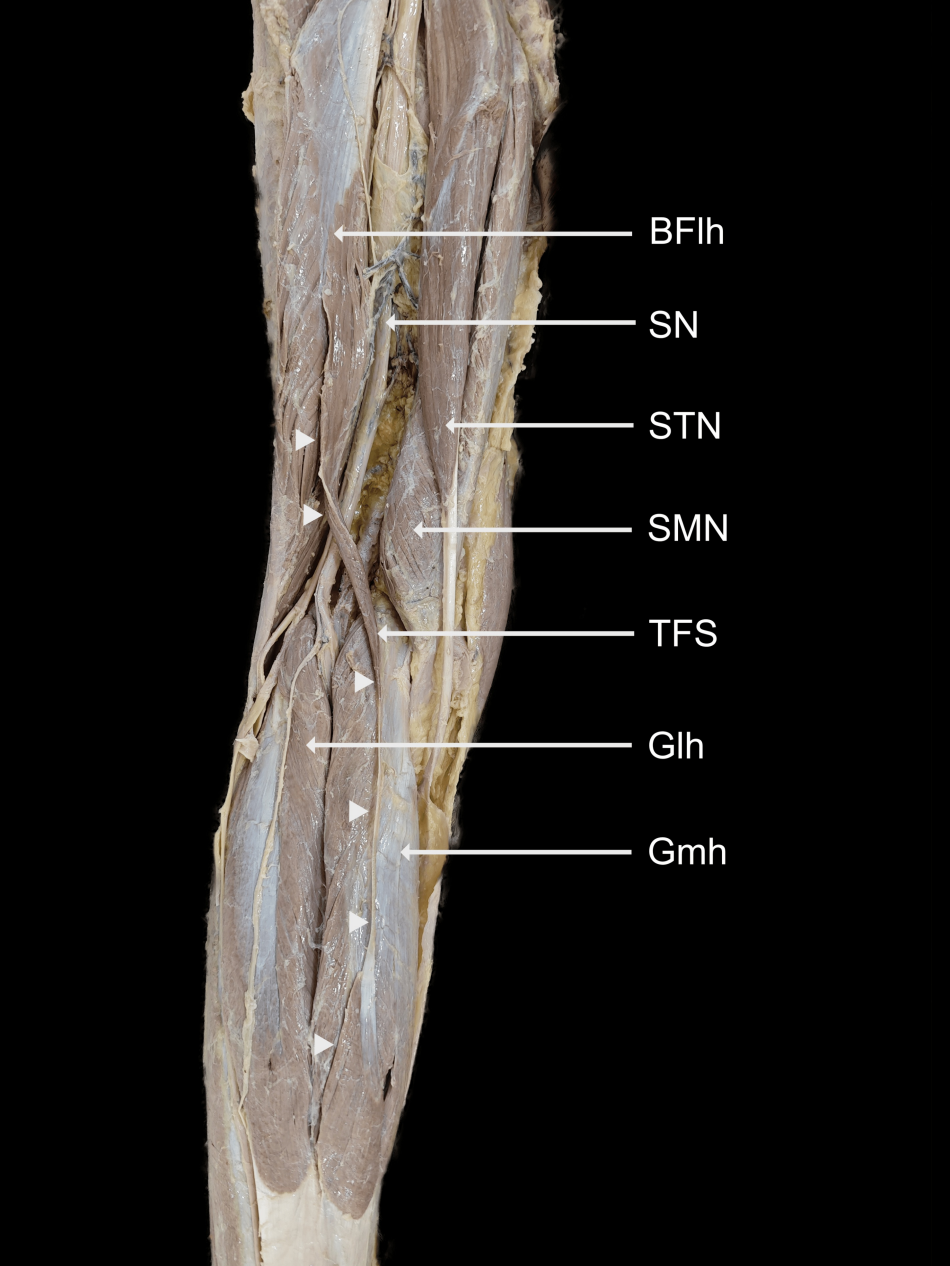
Dissected posterior compartments of the left lower limb. The TFS muscle can be seen originating from the long head of the biceps femoris and descending to terminate on the medial head of the gastrocnemius White arrowheads show the course of the TFS TFS, tensor fascia suralis; BFlh, long head of biceps femoris; SN, sciatic nerve; STN, semitendinosus; SMN, semimembranosus; Gmh, medial head of gastrocnemius; Glh, lateral head of gastrocnemius

**Figure 2:**
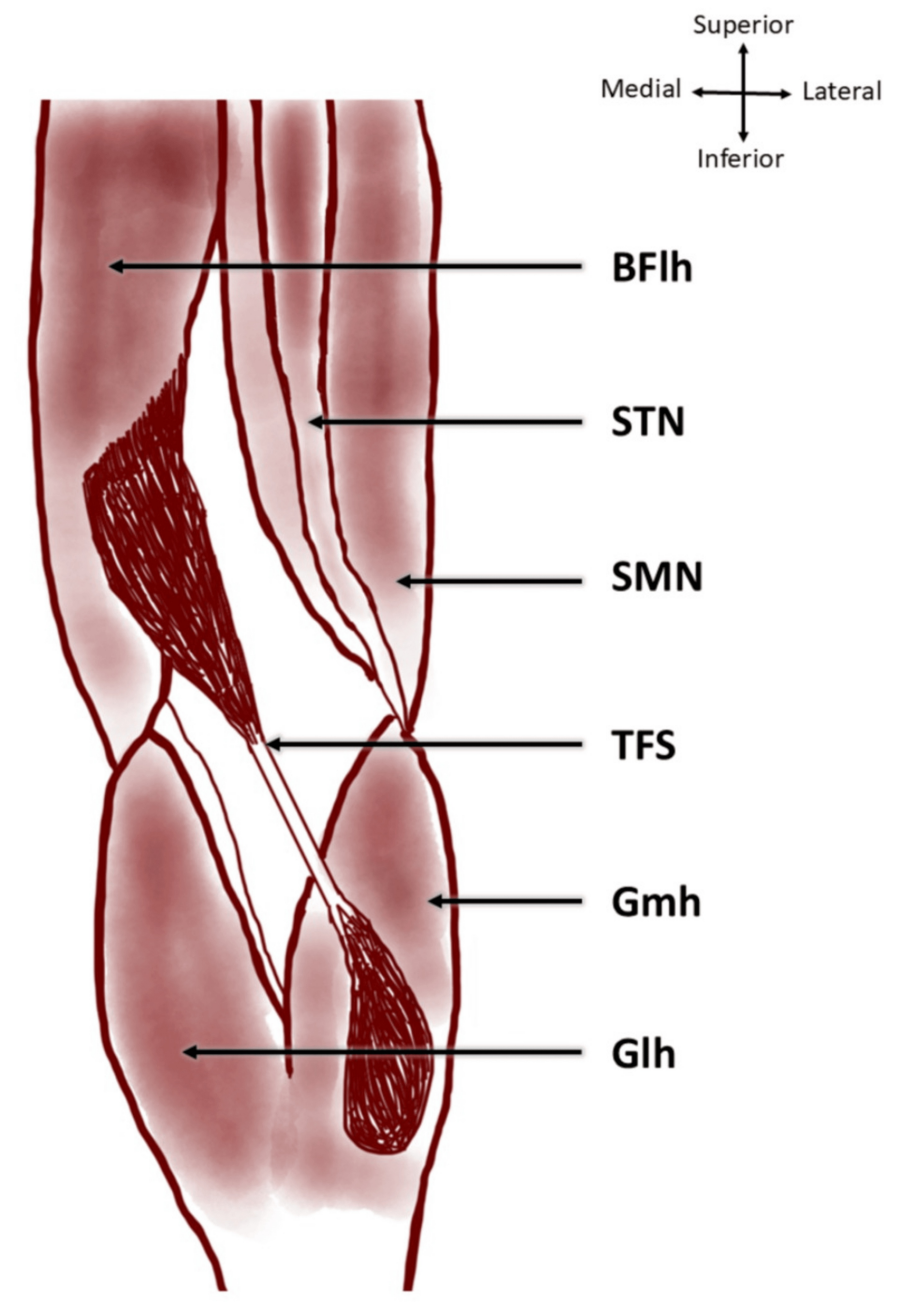
The TFS muscle originating from the long head of the biceps femoris and terminating on the medial head of gastrocnemius TFS, tensor fascia suralis; BFlh, long head of biceps femoris; STN, semitendinosus; SMN, semimembranosus; Gmh, medial head of gastrocnemius; Glh, lateral head of gastrocnemius Image credit: This is an original image created by the author Sayan Biswas

During the routine dissection of an Indian male cadaver, we identified aberrant muscular fibers in the left lower limb, extending between the long head of the biceps femoris and the insertion of the gastrocnemius tendon (Figures 1, 2).

**Figure 3:**
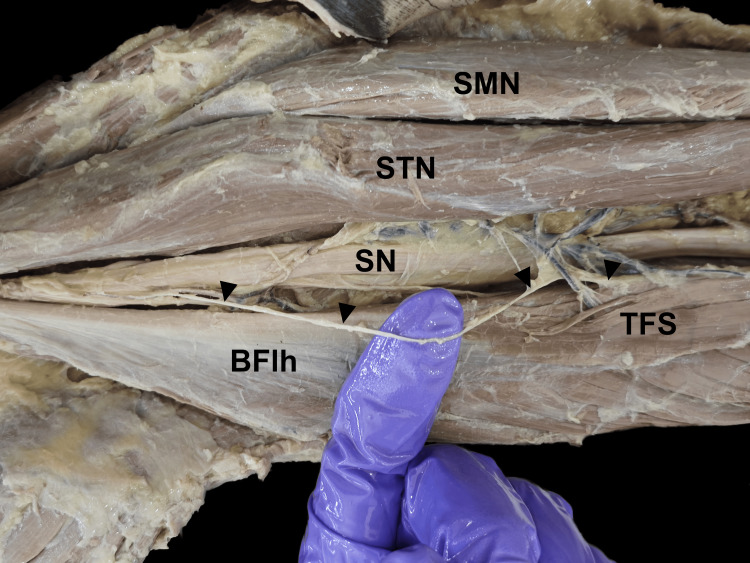
The upper belly of the TFS receiving innervation from a branch of the sciatic nerve (black arrowheads) TFS, tensor fascia suralis; SN, sciatic nerve; BFlh, long head of biceps femoris; STN, semitendinosus; SMN, semimembranosus

**Figure 4:**
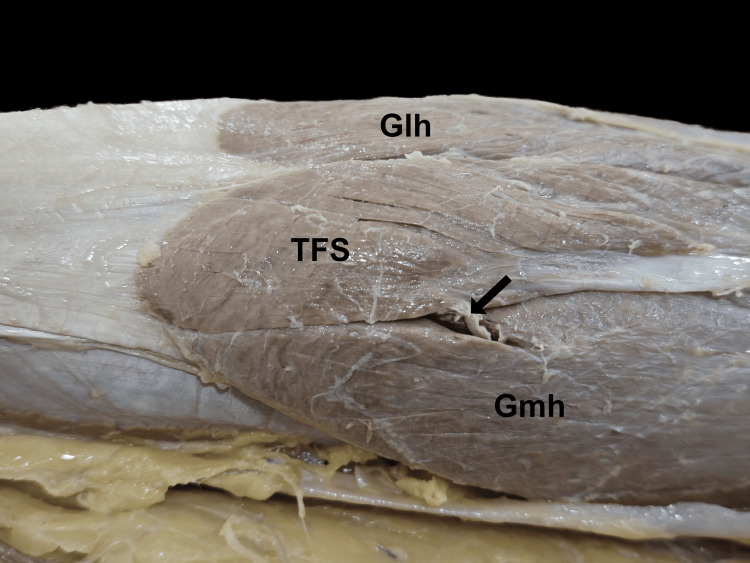
The lower belly of the TFS inserted into the tendon of the medial head of the gastrocnemius and received nerve supply from a branch of the tibial nerve that pierces through the medial head of the gastrocnemius muscle belly The black arrow shows the nerve supply TFS, tensor fascia suralis; Gmh, medial head of gastrocnemius; Glh, lateral head of gastrocnemius

From the biceps femoris, the muscle belly descended inferomedially as a common tendon over semitendinosus, enclosed in a loose sheath. Dissection revealed that this tendon was separate from the semitendinosus. It descended further down the back of the leg, forming a spindle-shaped muscular slip, which was broad and flattened in the anteroposterior aspect. It terminated by merging with the tendon of the medial head of the gastrocnemius muscle. The upper belly originated at a distance of 16 cm from the ischial tuberosity and measured 20 cm in length and 2.3 cm in width. The intermediate tendon was 11 cm long, 1.2 mm wide, and 0.6 mm thick, while the lower belly was observed to be 8.5 cm in length and 2.5 cm in width. The measurements were taken using a calibrated steel tape and a digital vernier calliper. The upper belly received innervation from a branch carrying the tibial component of the sciatic nerve above the popliteal fossa (Figure 3), whereas the lower belly was innervated by a branch of the tibial nerve after it pierced through the medial head of the gastrocnemius (Figure 4). There were no anatomical variations in the contralateral limb.

## Discussion

The presence of the TFS is a rare anatomical variant, with a prevalence rate of only 1.27%. Various studies have described muscular anomalies in the posterior compartment of the thigh and leg, attributing them to evolutionary remnants or adaptations for additional muscle function. First appearing in the literature in 1813, a proper classification system for TFS was developed more than two centuries later [2].

Although the insertion can vary, the TFS has been classified by Bale et al. into three types: type I where it originates from one of the hamstring muscles (Ia: long head of biceps femoris, Ib: semitendinosus, Ic: short head of biceps femoris, and Id: semimembranosus), type II where it originates in the form of double head from semitendinosus and biceps femoris, and type III comprising of all other variations. This case represents a variant of type Ia as it originates from the long head of the biceps femoris. These variants originate or insert as aponeurotic or tendinous structures with the muscle belly lying either at the origin or in the middle [2,3]. It consists entirely of muscle belly in cases where muscle fibers are transversely oriented [4]. Other variants include origin from the femur [1,5] or from the semimembranosus muscle [6]. An extensive literature search showed no more than 49 cases of TFS being published to date (Table 1), a summary of which has been provided in Table 2. The most common variant of this muscle was type Ia. It was mostly found in men and was innervated mainly by the tibial nerve.

**Table 1:** Case reports of tensor fascia suralis in the literature ^*^Radiological case reports. Others are reported after gross dissection Subtypes are according to the classification by Bale et al. [2] on the basis of the origin of the tensor fascia suralis M: male; F: female; TN: tibial nerve; CPN: common peroneal nerve; Types Ia: origin from long head of biceps femoris; Ib: semitendinosus; Ic: short head of biceps femoris; II: double heads with medial head from semitendinosus and lateral head from long head of biceps femoris; III: all other variations

Sl. no.	Year	Author	Side	Sex	Subtype	Nerve	Reference
1	1813	Kelch	-	-	Ia	-	[2]
2	1870	Gruber	-	-	Ib	-	[7]
3	1872	Turner	Right	-	Ib	-	[7]
4	1872	Turner	-	-	Ia	-	[7]
5	1873	Gruber	Left	M	Ia	-	[7]
6	1873	Gruber	Right	M	Ib	-	[7]
7	1873	Gruber	Left	M	Ib	-	[7]
8	1884	Turner	-	-	Ia	-	[7]
9	1879	Gruber	Left	M	Ic	-	[7]
10	1879	Gruber	Left	M	Ia	-	[7]
11	1881	Halliburton	Right	F	III	-	[2]
12	1897	Gruber	-	-	Ia	-	[7]
13	1911	Klaatsch	Right	M	Ic	TN	[2]
14	1913	Schaeffer	Left	M	Ia	-	[2]
15	1924	Barry and Bothroyd	Left	M	III	TN	[3]
16	1935	Kawai	-	-	Ib	-	[7]
17	1940	Mogi	Right	M	Ib	TN	[2]
18	1954	Nonaka and Ishii	-	-	II	-	[2]
19	1985	Miyauchi et al.	Right	M	II	-	[2]
20	1995	Chason et al.^*^	-	-	Ib	-	[7]
21	1995	Sinav et al.	Bilateral	F	Ia	TN	[2]
22	1998	Somayaji et al.	-	-	II	TN	[2]
23	2001	Seema and Balalkrishna	Right	-	III	-	[2]
24	2002	Montet et al.^*^	Right	M	Ib	-	[2]
25	2004	Okamoto et al.	Right	F	Ic	CPN	[4]
26	2006	Tubbs et al.	Right	M	Ib	TN	[2]
27	2006	Kumar	Right	M	II	TN	[8]
28	2009	Kim et al.	Right	F	Ia	CPN	[9]
29	2009	Luca et al.	Right	M	Ia	TN	[10]
30	2011	Padmalatha et al.	Left	M	Ib	-	[2]
31	2014	Feimster et al.	Bilateral	M	Ia	TN	[11]
32	2014	Sowmya et al.	Right	M	Ia	TN	[12]
33	2015	Gandhi et al.	Bilateral	M	Ia	TN	[2]
34	2015	Kim et al.^*^	Right	M	Ib	-	[2]
35	2016	Rajendiran and Murugesan	Left	M	Ia	TN	[13]
36	2017	Dickson and Koulouris^*^	Left	M	Ia	-	[14]
37	2017	Oommen et al.	Left	M	II	-	[7]
38	2017	Arakawa et al.	Right	M	Ia	TN	[15]
39	2017	Bale and Herrin	Bilateral	M	Ia	-	[2]
40	2017	Tsifountoudis et al.^*^	Right	M	Ib	-	[16]
41	2018	Elliott	Right	M	Ib	-	[7]
42	2019	George et al.	Left	M	III	CPN	[5]
43	2020	Boudier-Revéret et al.^*^	Bilateral	M	Ib	-	[7]
44	2021	Olewnik et al.	Right	M	III	-	[1]
45	2021	Olateju	Left	M	Ia	TN	[17]
46	2023	Bale et al.	Left	M	Ia	-	[2]
47	2024	Swancutt et al.	Bilateral	F	Ia	-	[18]
48	2024	Snow et al.	Left	-	Ia	-	[7]
49	2025	Lee et al.^*^	Right	M	Id	-	[6]
50	2025	Present case report	Left	M	Ia	TN	-

**Table 2:** Summary of the case reports of tensor fascia suralis in the literature (total number of cases: 50 including current study) Subtypes are according to the classification by Bale et al. [2] on the basis of the origin of the tensor fascia suralis Types Ia: origin from long head of biceps femoris; Ib: semitendinosus; Ic: short head of biceps femoris; II: double heads with medial head from semitendinosus and lateral head from long head of biceps femoris; III: all other variations

Parameters	Number of cases	Reference
Study types	Gross dissection: 43	[2-5,7-13,15,17,18]
	Radiological: 7	[2,6,7,14,16]
Side	Right: 20	[2,7-10,12,15,16]
	Left: 15	[2,3,5,7,13,14,17]
	Bilateral: 6	[2,7,11,18]
	Unknown: 9	[2,7]
Sex	Male: 33	[2,3,7,8,10-17]
	Female: 5	[2,4,9,18]
	Unknown: 12	[2,7]
Innervation	Tibial nerve: 15	[2,3,8,10-13,15,17]
	Common peroneal nerve: 3	[4,5,9]
	Unknown: 32	[2,7,14,16,18]
Subtypes	Ia: 22	[2,7,9-15,17,18]
	Ib: 14	[2,7,16]
	Ic: 3	[2,4,7]
	Id: 1	[6]
	II: 5	[2,7,8]
	III: 5	[1,2,3,5]

This case is unique in that it features a sequence of a muscle belly, an intermediate tendon, followed by the reforming of a flattened muscular slip. Although double muscle bellies have been seen in TFS, they both lie at the origin, unlike at either end, as observed in the present case [8]. The only case having two bellies separated by an intermediate tendon was reported by Olateju [17]. However, the proximal belly had a commonly observed tendinous origin, unlike the fleshy origin in our case. Moreover, both bellies in this case were supplied by tibial nerve components, but the upper belly from the sciatic nerve directly and the lower one after the sciatic nerve had divided in the popliteal fossa.

During development, the ventral muscle mass of the lower limb buds gives rise to the posterior compartment muscles of both thigh and leg under the influence of Shh signaling [19]. Hence, although the embryological basis of TFS is not well established, it is thought to be due to the failure of the disappearance of the embryological fibrous connection that spans the deep fascia of the leg and the biceps femoris tendon [2]. It may also develop as a remnant slip during embryological division of the knee flexor muscle group into individual hamstring muscles [7]. Thus, TFS may be seen with other anomalies in lower limb musculature [15,18]. It is also akin to some muscles found in certain mammals, an example being the abductor cruris caudalis seen in dogs and cats [20].

From a clinical perspective, the presence of this muscle may pose diagnostic challenges. It may be mistaken for soft tissue tumors or abnormal muscle hypertrophy in radiological investigations such as MRI and ultrasound scans, or during clinical examination. Additionally, as the TFS is located near key structures like the sciatic nerve, popliteal vessels, hamstrings, and gastrocnemius, it may lead to compression syndromes and contribute to motor and sensory impairments in the lower leg and foot, necessitating careful evaluation by clinicians [6,9]. Surgeons performing procedures in the posterior compartment of the thigh and leg should be aware of such variations to prevent inadvertent injuries, particularly during hamstring graft harvesting or reconstructive surgeries.

Functionally, the TFS may contribute to biomechanical movements of the lower limb, particularly in assisting flexion at the knee and plantarflexion at the ankle. Biomechanical analysis by Snow et al. observed that the maximal isometric force stood at 8.10 N, thus assisting the surrounding popliteal muscles, which have 9-27 times the force [7]. Athletes presenting with unexplained posterior thigh pain or stiffness may benefit from imaging and physical examination, considering the possibility of an accessory muscle structure. Targeted physiotherapy and myofascial release techniques may help in alleviating symptoms in such cases [14].

## Conclusions

TFS having two muscle bellies without any tendinous structure on either end has not been reported before. It is a rare but clinically significant muscle, especially in posterior thigh and knee surgeries. Recognizing its variant morphology is essential for preventing nerve damage and ensuring accurate clinical and radiological diagnosis and surgical outcomes.
